# A random forest algorithm-based prediction model for moderate to severe acute postoperative pain after orthopedic surgery under general anesthesia

**DOI:** 10.1186/s12871-023-02328-1

**Published:** 2023-11-06

**Authors:** Gaoxiang Shi, Geliang Liu, Qichao Gao, Shengxiao Zhang, Qi Wang, Li Wu, Peifeng He, Qi Yu

**Affiliations:** 1 School of Basic Medical Sciences, Shanxi Medical University, Taiyuan, China; 2 Institute of Medical Data Science, Shanxi Medical University, Taiyuan, China; 3 Shanxi Key Laboratory of Big Data for Clinical Decision, Shanxi Medical University, Taiyuan, China; 4 Department of Anesthesiology, Shanxi Bethune Hospital, Shanxi Academy of Medical Sciences, Tongji Shanxi Hospital, Third Hospital of Shanxi Medical University, Taiyuan, China; 5 School of Management, Shanxi Medical University, Taiyuan, China; 6 Department of Rheumatology, Second Hospital of Shanxi Medical University, Taiyuan, China; 7 Key Laboratory of Cellular Physiology, Ministry of Education, Shanxi Medical University, Taiyuan, China

**Keywords:** Random forest model, Logistic regression model, Machine learning, Pain prediction, Analgesia

## Abstract

**Background:**

Postoperative pain is one of the most common complications after surgery. In order to detect early and intervene in time for moderate to severe postoperative pain, it is necessary to identify risk factors and construct clinical prediction models. This study aimed to identify significant risk factors and establish a better-performing model to predict moderate to severe acute postoperative pain after orthopedic surgery under general anesthesia.

**Methods:**

Patients who underwent orthopedic surgery under general anesthesia were divided into patients with moderate to severe pain group (group P) and patients without moderate to severe pain group (group N) based on VAS scores. The features selected by Lasso regression were processed by the random forest and multivariate logistic regression models to predict pain outcomes. The classification performance of the two models was evaluated through the testing set. The area under the curves (AUC), the accuracy of the classifiers, and the classification error rate for both classifiers were calculated, the better-performing model was used to predict moderate to severe acute postoperative pain after orthopedic surgery under general anesthesia.

**Results:**

A total of 327 patients were enrolled in this study (228 in the training set and 99 in the testing set). The incidence of moderate to severe postoperative pain was 41.3%. The random forest model revealed a classification error rate of 25.2% and an AUC of 0.810 in the testing set. The multivariate logistic regression model revealed a classification error rate of 31.3% and an AUC of 0.764 in the testing set. The random forest model was chosen for predicting clinical outcomes in this study. The risk factors with the greatest and second contribution were immobilization and duration of surgery, respectively.

**Conclusions:**

The random forest model can be used to predict moderate to severe acute postoperative pain after orthopedic surgery under general anesthesia, which is of potential clinical application value.

## Background

Postoperative pain is one of the most common complications after surgery, the incidence of moderate to severe postoperative pain varies from 25 to 66% according to the previous reports [1, 2]. The consequences of suboptimal postoperative pain control include negative effects on postoperative recovery, increased incidence of respiratory and circulatory complications, increased length of hospital stay and healthcare costs, as well as an increased risk of transition to chronic pain or neuropathic pain [3, 4]. With the advances in modern medicine, postoperative pain remains a challenge, hence improving pain control is an international initiative promoted by multiple health organizations including WHO [5].

Orthopedic surgeries are considered to be some of the most painful procedures that have a variety of options for postoperative analgesia ranging from surgeon provided (e.g., local anesthesia) to more intensive techniques (e.g., nerve blockade or patient-controlled epidural analgesia) requiring care from an acute pain service [6]. According to the author’s clinical experience and some research reports, compared with patients receiving spinal anesthesia or regional anesthesia, acute postoperative pain is more severe in patients undergoing orthopedic surgery under general anesthesia [7, 8].

The ability to identify and focus care on patients at higher risk of moderate to severe postoperative pain would improve analgesia and patient satisfaction. The construction of a reliable postoperative pain prediction model based on risk factors can be applied in the early identification of orthopedic patients with a high risk of moderate to severe postoperative pain, which is vital in taking timely interventions to prevent pain from worsening.

Random forest algorithms can build a machine learning model based on sample data and be used to make predictions, and its performance advantages are mainly due to ensemble learning [9]. The previous studies demonstrated that the logistic regression model had limited performance in predicting acute postoperative pain [8, 10], while there have been no reports of using random forests to predict postoperative pain to the best of our knowledge.

Accordingly, we constructed machine learning models to predict moderate to severe acute postoperative pain of orthopedic patients under general anesthesia by identifying the risk factors. In addition, we evaluated the efficiency of the random forest algorithm-based prediction model by comparing it with the multivariate logistic regression-based model.

## Materials and methods

**Fig. 1:**
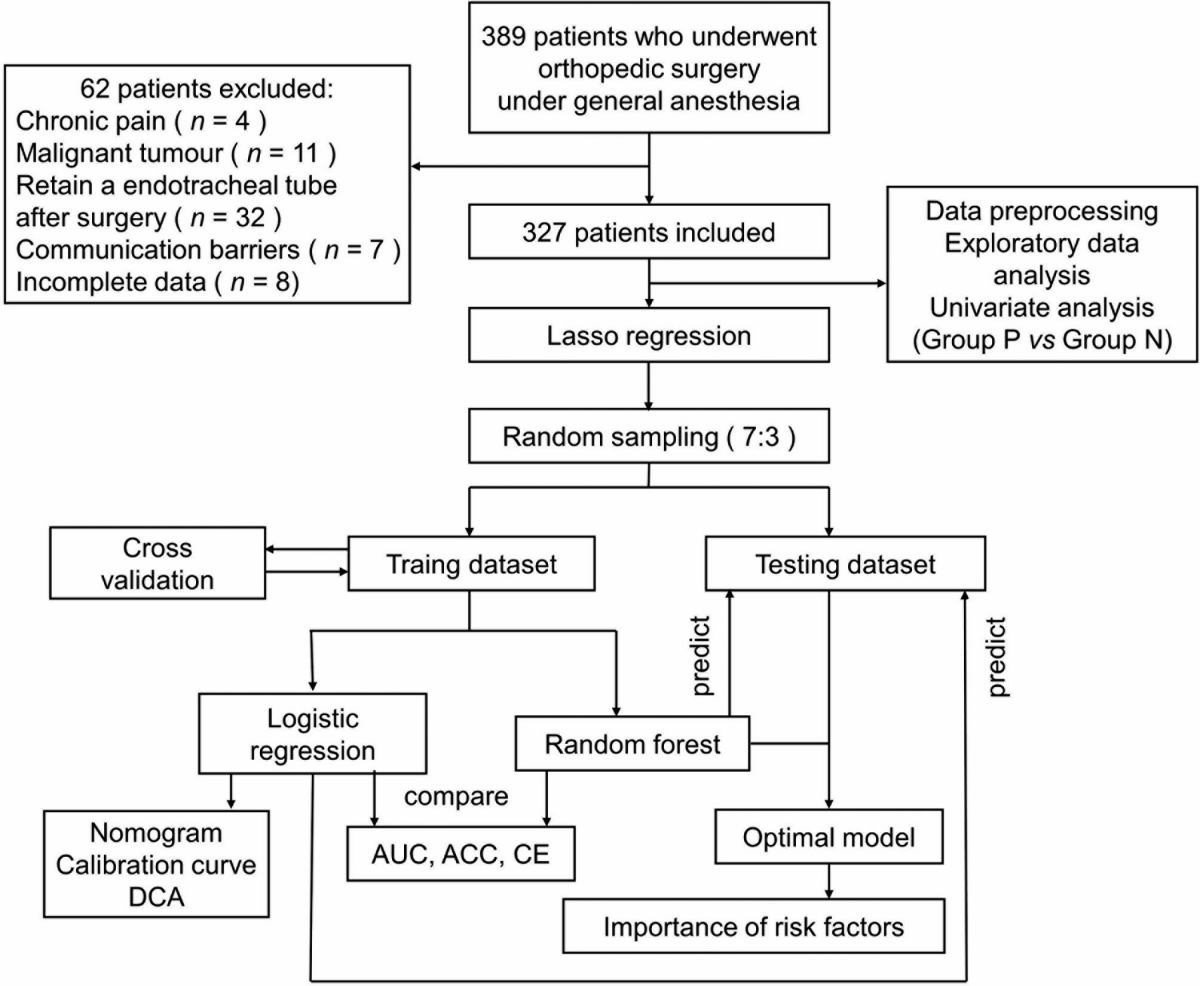
The procedure of establishing moderate to severe acute postoperative pain prediction models in this study

This retrospective observational cohort study was conducted following the Declaration of Helsinki (as revised in October 2013). The study was approved and monitored by the Ethics Committee of Shanxi Bethune Hospital (Third Hospital of Shanxi Medical University). Because of the retrospective nature of the study and the patient’s identity information has been concealed, the requirement for informed consent was waived by the Ethics Committee of Shanxi Bethune Hospital. We present the following article in accordance with the Transparent Reporting of a Multivariable Prediction Model for Individual Prognosis or Diagnosis (TRIPOD) reporting checklist [11]. The procedure of establishing moderate to severe acute postoperative pain prediction models in this study is shown in Fig. 1.

### Sample selection

Patients who underwent orthopedic surgery under general anesthesia in Shanxi Bethune Hospital from January 2020 to June 2020, were included in the study. The demographic and perioperative characteristics were extracted from the Electronic Medical Record (EMR) database.

### Inclusion criteria

The inclusion criteria for this study were as follows: (1) Patients between the age of 18 and 100. (2) Patients underwent orthopedic surgery under general anesthesia. Children and adolescents were not included in this study because they are in the stage of growth and development, and their physiological characteristics are more complex, so they are not suitable for study together with adults.

### Exclusion criteria

The exclusion criteria for this study were as follows: (1) Patients with chronic pain (which included musculoskeletal pain disorders, peripheral neuropathy, and migraines), (2) Patients with malignant tumors. Because long-term chronic pain such as tumors is prone to special conditions such as hyperalgesia and neuropathic pain, resulting in inaccurate pain scores. (3) Patients retain an endotracheal tube after surgery, (4) Patients with cognitive dysfunction or who cannot communicate normally. Due to the difficulty of self-assessing pain scores in these two groups. (5) Outpatient surgeries, (6) Incomplete clinical data. Because the medical records in these two groups may miss key data.

### Pain scoring methods and diagnosis of moderate to severe postoperative pain

The primary outcome was pain scores at rest on postoperative day one (POD1) using a visual analogue scale (VAS), with 0 representing no pain and 10 representing the most intense pain. Moderate to severe pain was defined as a VAS score of 4 or greater, which has been previously identified as a value at which patients request additional analgesias, become unsatisfied with pain control, and have interference with functional activity [12]. The VAS score was self-assessed by the patients based on his or her pain level under the guidance of an anesthesiologist or anesthesia nurse, and recorded by the anesthesiologist. Patients and staff were blinded to this study.

### Variables

Demographic variables were defined and analyzed as follows: sex, age, and body mass index (BMI), which have been shown to be associated with postoperative pain in many studies [13–15]. Perioperative variables including physical status score based on the American Society of Anesthesiologists physical status classification (ASA score), which was routinely included in anesthesia-related studies [16]. surgical score, type of surgery (open surgery vs. endoscopic surgery), surgical site (spinal area, joint, limb bones, muscles and soft tissues), blood loss during surgery, intraoperative blood transfusion, indwelling urinary catheters, indwelling drains, tourniquet during surgery, and arteriovenous catheterization were included to reflect the degree of tissue damage and intensity of noxious stimulation [17, 18]. Multimodal analgesia methods contained patient-controlled intravenous analgesia (PCIA) pumps, peripheral nerve blockade, and preemptive analgesia were included, which may be beneficial in reducing the incidence of acute postoperative pain [19, 20]. Variables including history of surgery or anesthesia, history of depression or anxiety, preoperative VAS score, immobilization, secondary surgery in a short period (within a month), and timing of surgery (emergency surgery vs. elective surgery) may reflect special medical history related to postoperative pain [21–23]. Furthermore, the duration of surgery, time from withdrawal of medicine to awake, consumption of sufentanil, remifentanil, propofol, sevoflurane and rocuronium were included to assess the impact of drug dosage, time and other factors on outcomes [24, 25]. In particular, “surgical score” is a scoring system developed by the National Health Commission of the PRC according to the difficulty and risk of surgery, with a score ranging from 1 to 4, the higher scores indicating greater surgical difficulty. And “arteriovenous catheterization” refers to puncture catheterization for the purpose of invasive blood pressure measurement or infusion through the central venous.

### Feature selection

The pre-processed data were randomly split into training and testing sets. In the training set, demographic and perioperative characteristics above were selected as candidate risk factors because of previous reports and clinical experiences. After univariate analysis, the Lasso regression model was applied to screen the optimized variables by running cyclic coordinate descent. Age, duration of surgery, blood loss during surgery, time from withdrawal of medicine to awake, sufentanil consumption, remifentanil consumption, propofol consumption, sevoflurane consumption, and rocuronium consumption were entered into the Lasso regression procedure as continuous variables. ASA score, timing of surgery, type of surgery, indwelling urinary catheters, arteriovenous catheterization, secondary surgery in a short period, immobilization, intraoperative blood transfusion, and tourniquet during surgery were entered as dichotomous variables. Lasso regression was generated using the glmnet package in R, the optimal lambda value was determined by 10-fold cross-validation. Lasso regression can force the coefficients of redundant variables to 0 and thus directly exclude them. The retained variables were selected as the input variables of the random forest models and multivariate logistic regression models.

### Random forest modeling

The mlr3 package based on R was applied for random forest model construction and hyperparameter tuning. The data was resampled by using the bootstrapping/bagging method. The variation range of the hyperparameter space were pre-set as: “num.trees” [300 ~ 1000], “mtry” [2 ~ 5], “min.node.size” [2 ~ 10], and “max.depth” [2 ~ 10]. AutoTuner functions of the mlr3 package were used for the grid search and automatic tuning of hyperparameters, the cross-validation technique was used to tune the number of estimators in the classifier, and all training was conducted with 10-fold cross-validation to prevent overfitting. All the indicators included in the risk prediction model were analyzed based on the mean decrease in accuracy and the mean decrease in the Gini coefficient.

### Logistic regression modeling

The mlr3 package was applied for logistic regression model construction and hyperparameter tuning. The training set was conducted with 10-fold cross-validation to improve predictive performance and prevent overfitting. Independent risk factors were identified using a multivariate logistic regression model that entered variables selected in Lasso analysis, and odds ratio (OR) along with 95% confidence interval (CI) were calculated. The nomograms were applied to visualize the prediction model, the calibration curves were applied to visualize Hosmer-Lemeshow goodness-of-fit test, and the decision curves were used to determine clinical benefit.

### Evaluation of machine learning models

The confusion matrixes, the accuracy of the classifiers (“classif.acc”), the classification error rate (“classif.ce”) and the area under the receiver operating characteristic curve (AUC) were analyzed to evaluate the performance and clinical usefulness of the random forest classifier and the logistic regression classifier by comparing the predicted results with the true results. Given that the incidence of positive events in this study was 41.3%, the threshold of the ROC curve was set to 0.4 instead of the default 0.5.

### Statistical analysis

Statistical analyses were performed using the RStudio software (version 2022.12.0-353), which runs R software (version 4.1.3; http://www.Rproject.org). Descriptive statistics were computed for all variables. These included means and standard deviations (SD) for continuous variables that conform to normal distributions, median and interquartile range for continuous variables that do not conform to normal distributions, and frequencies for categorical factors. Comparisons of the distribution of demographic variables and clinical characteristics were performed using the two-tailed t-test (or the Mann-Whitney test as appropriate) for continuous variables and the chi-square test (or the Fisher exact test as appropriate) for categorical variables. P values of 0.05 or lower were considered statistically significant.

## Results

### Patient characteristics

A total of 327 patients were enrolled in this study, The rate of moderate to severe acute postoperative pain among all enrolled patients was 41.3%. After univariate analysis, fourteen characteristics were retained for subsequent Lasso analysis. The demographic and perioperative characteristics of all enrolled patients are shown in Table 1.

**Table 1:** **The demographic and perioperative characteristics of all enrolled patients**

Variables	Total patients (*n* = 327)	Group P (*n* = 135)	Group N (*n* = 192)	*t* / χ^2^ / *Z*	*P*-value
**Demographics**					
Sex				0.3378	0.5611
Male	201	86	115		
Female	126	49	77		
Age, years	51.63 ± 14.38	48.47 ± 14.14	53.84 ± 14.16	3.3781	0.0008
BMI, kg/m^2^	24.66 ± 3.92	24.27 ± 4.12	24.93 ± 3.76	1.4772	0.1408
**Perioperatives**					
ASA score				8.8704	0.0029
I or II	218	77	141		
III or IV	109	58	51		
Surgical score				0.55496	0.4563
I or II	35	17	18		
III or IV	292	118	174		
Timing of surgery				11.634	0.0006
emergency surgery	62	38	24		
elective surgery	265	97	168		
Type of surgery				4.7037	0.0301
open surgery	289	126	163		
endoscopic surgery	38	9	29		
Surgical site				6.5915	0.0861
spinal area	130	59	71		
joint	51	24	27		
limb bones	90	27	63		
muscles and soft tissues	56	25	31		
History of surgery or anesthesia				0.5876	0.4434
yes	86	32	54		
no	241	103	138		
Preoperative VAS score	0 (0-0)	0 (1-0)	0 (0-0)	-0.8729	0.3827
History of depression or anxiety				2.3131	0.1283
yes	12	8	4		
no	315	127	188		
Indwelling urinary catheters				14.806	0.0001
yes	227	110	117		
no	100	25	75		
Indwelling drains				2.2149	0.1367
yes	300	128	172		
no	27	7	20		
PCIA				2.444	0.118
yes	272	118	154		
no	55	17	38		
Nerve blockade				0.7377	0.3904
yes	49	17	32		
no	278	118	160		
Arteriovenous catheterization				14.299	0.0002
yes	80	48	32		
no	247	87	160		
Secondary surgery in a short period				4.9972	0.0253
yes	19	13	6		
no	308	122	186		
Duration of surgery, minutes	124 (170-80)	135 (185-104.5)	110 (159.25-72)	11.0483	7.286e-05
Blood loss during surgery, ml	150 (400-100)	200 (475-100)	150 (300-57.5)	11.1878	9.011e-05
Time from withdrawal of medicine to awake, minutes	15.77 ± 3.76	15.10 ± 3.81	16.24 ± 3.66	2.7464	0.006
Preemptive analgesia				0.21917	0.6397
yes	243	98	145		
no	84	37	47		
Immobilization				29.615	5.27e-08
yes	104	66	38		
no	223	69	154		
Intraoperative blood transfusion				10.794	0.0010
yes	95	53	42		
no	232	82	150		
Sufentanil consumption, µg	45 (50-40)	50 (60-40)	40 (50-35)	11.8357	0.0003
Remifentanil consumption, mg	1.2 (1.5-0.8)	1.2 (1.7-1)	1 (1.5-0.7)	-3.2160	0.0013
Propofol consumption, mg	700 (875-475)	700 (900-500)	575 (850-400)	-3.0314	0.0024
Sevoflurane consumption, ml	30 (40-15)	30 (45-22.5)	25 (40-15)	-3.7777	0.0002
Rocuronium consumption, mg	90 (120-65)	100 (125-70)	80 (120-57.5)	-2.9820	0.0029
Tourniquet during surgery				52.177	5.071e-13
yes	65	53	12		
no	262	82	180		

### Filtered features for machine learning model establishing

**Fig. 2:**
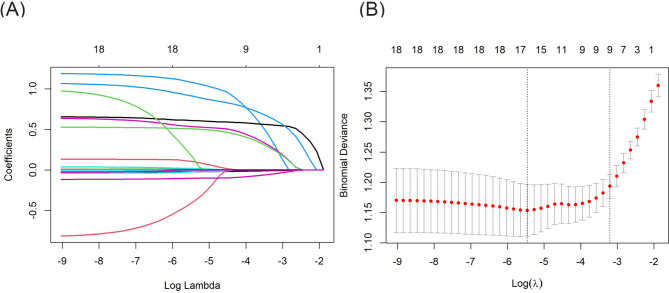
**(A)** Lasso coefficient profiles of all candidate features. **(B)** The tuning parameter λ (lambda) selection in the Lasso models used 10-fold cross-validation by minimum criteria

Using the Lasso regression model, eighteen characteristics were tested for their ability to predict the clinical outcomes and to avoid overfitting. The Lasso coefficient profiles of features and the optimal penalization coefficient lambda+1se are shown in Fig. 2. The feature selection results revealed that nine variables, including age, indwelling urinary catheters, arteriovenous catheterization, secondary surgery in a short period of time, duration of surgery, blood loss during surgery, immobilization, time from withdrawal of medicine to awake and tourniquet during surgery, could be used to predict moderate to severe acute postoperative pain (Table 2).

**Table 2:** The characteristics of all enrolled patients(Training set vs. Testing set)

Variables	Total patients (*n* = 327)	Training set (*n* = 228)	Testing set (*n* = 99)	*t* / χ^2^ / *Z*	*P*-value
**Demographics**					
Age, years	51.63 ± 14.38	52.32 ± 13.74	50.03 ± 15.71	-1.2564	0.2107
**Perioperatives**					
Indwelling urinary catheters				0.9726	0.324
yes	227	154	73		
no	100	74	26		
Arteriovenous catheterization				0.84331	0.3585
yes	80	52	28		
no	247	176	71		
Secondary surgery in a short period				0.016849	0.8967
yes	19	14	5		
no	308	214	94		
Duration of surgery, minutes	124 (170-80)	124 (166.25-81.5)	113 (170-79)	2.1046	0.673
Blood loss during surgery, ml	150 (400-100)	150 (400-80)	200 (400-100)	1.3283	0.572
Time from withdrawal of medicine to awake, minutes	15.77 ± 3.76	15.82 ± 3.67	15.66 ± 3.97	-0.34078	0.7337
Immobilization				0.60667	0.436
yes	104	69	35		
no	223	159	64		
Tourniquet during surgery				4.2322	0.03966
yes	65	38	27		
no	262	190	72		
Moderate to severe postoperative pain				0.11243	0.7374
yes	135	96	39		
no	192	132	60		

### Random forest algorithm-based prediction model

**Fig. 3:**
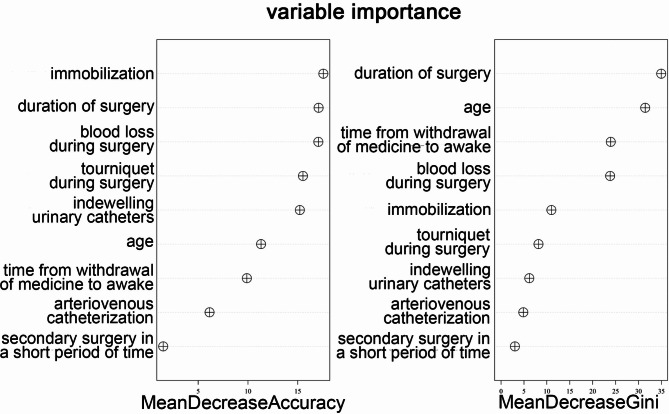
Importance of risk factors in the prediction model constructed by random forest

A risk prediction model was constructed based on confirmed characteristics selected by the Lasso algorithm. The number of decision trees was set at 500, the “mtry” parameter was set at 3, the “min.node.size” parameter was set at 5, and the “max.depth” parameter was set at 6 according to the cross-validation algorithm and AutoTuner function. As shown in Fig. 3, the mean decrease in accuracy and mean decrease in Gini for all indicators entered in the random forest model were analyzed. The mean decrease in accuracy showed that immobilization was the highest, followed by duration of surgery, blood loss during surgery, tourniquet during surgery, indwelling urinary catheters, etc. It refers to the degree of decrease in accuracy without the presence of this risk factor in the random forest model, which is equivalent to the classification contribution.

### Logistic regression algorithm-based prediction model

**Fig. 4:**
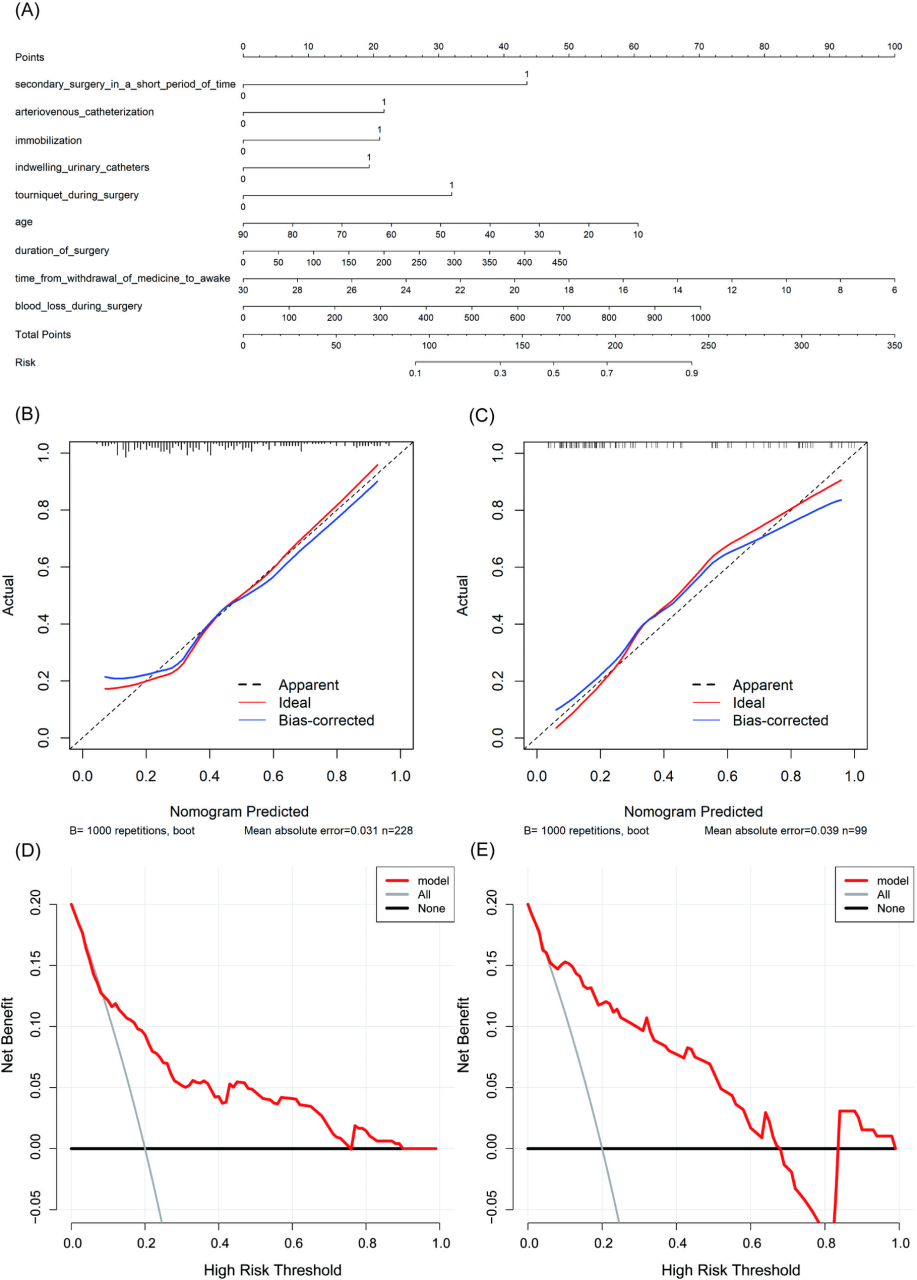
Visualization and performance evaluation of the predictive model based on multivariate logistic regression. **(A)** The nomogram. **(B)** The calibration curve in the training set. **(C)** The calibration curve in the testing set. **(D)** The decision curve in the training set. **(E)** The decision curve in the testing set

To verify the efficacy of the random forest model, we constructed a multivariate logistic regression model to predict moderate to severe acute postoperative pain, the model is visualized in Fig. 4A. Based on the multivariate analysis, three characteristics, namely shorter time from withdrawal of medicine to awake [OR 1.19, 95% CI (1.08, 1.31)], immobilization [OR 2.36, 95% CI (1.15, 4.85)], and indwelling urinary catheters [OR 2.39, 95% CI (1.09, 5.27)] were identified as independent risk factors. As shown in Fig. 4**(B and C)**, the calibration plots showed favorable consistency between the prediction of the logistic model and actual observations in both the training and testing sets. Furthermore, As shown in Fig. 4** (D and E)**, the DCA plots showed that the logistic model was clinically useful and had good predictive ability in the training set.

### Evaluation of predictor performance

**Fig. 5:**
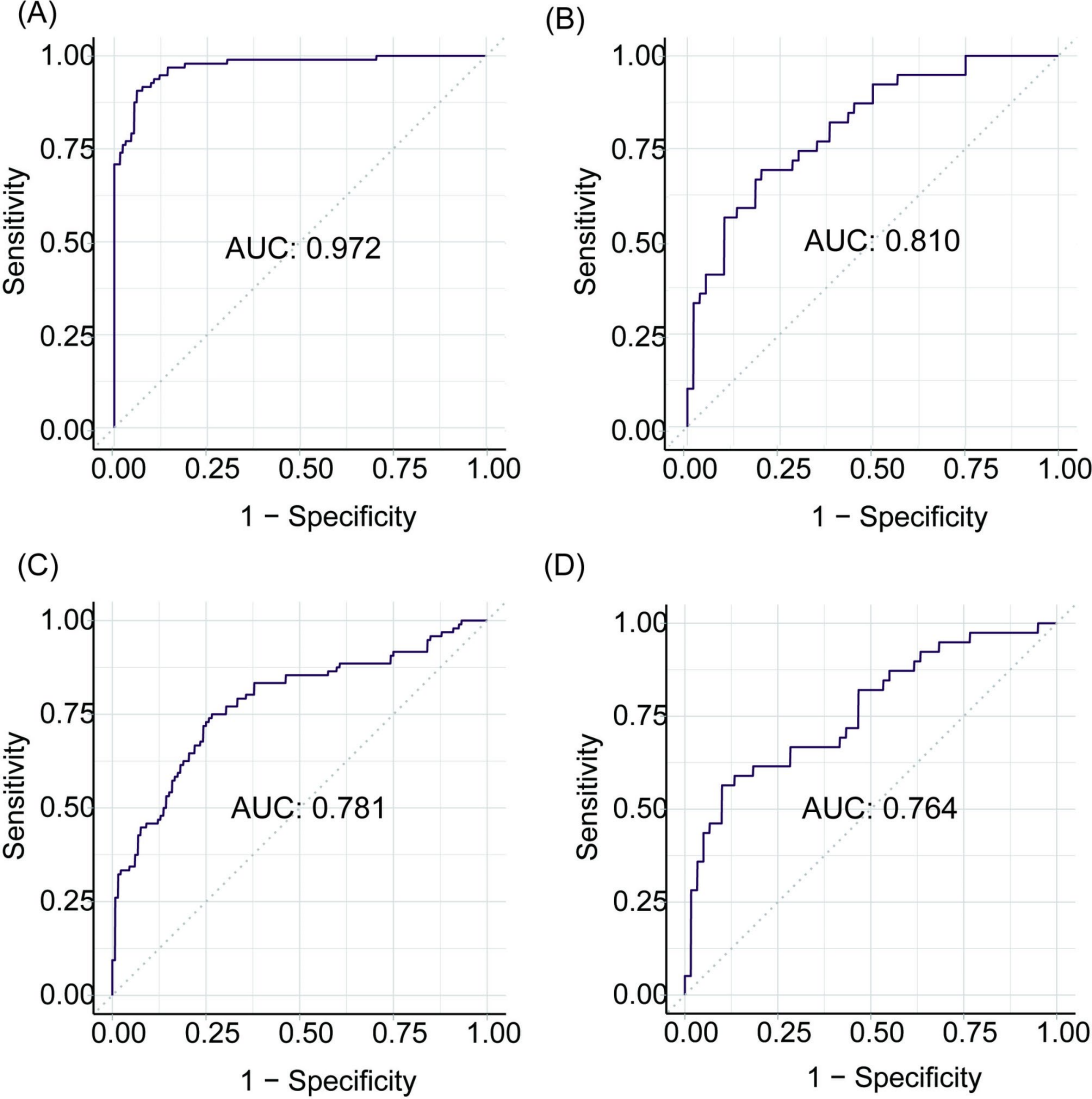
The ROC curves of the models in this study. **(A)** The ROC curve of the prediction model constructed by random forest in the training set. **(B)** The ROC curve of the prediction model constructed by random forest in the testing set. **(C)** The ROC curve of the prediction model constructed by multivariate logistic regression in the training set. **(D)** The ROC curve of the prediction model constructed by multivariate logistic regression in the testing set

The ROC curves of prediction models constructed by random forest and traditional logistic regression in the training and testing sets are shown in Fig. 5. The AUC of the random forest algorithm-based prediction model in the training and testing sets were 0.972 and 0.810, respectively, which confirmed the good discrimination performance of the prediction model. Additionally, the AUC of the risk prediction model constructed by multivariate logistic regression in the training and testing sets were 0.781 and 0.764, respectively.

The accuracy and the error rate were applied in testing the reliability of prediction models in our study. The accuracy values of the random forest algorithm-based prediction model and multivariate logistic regression-based prediction model in the training set were 0.882 and 0.724, respectively. The accuracy values of the random forest algorithm-based prediction model and logistic regression-based prediction model in the testing set were 0.747 and 0.687, respectively.

## Discussion

Despite extraordinary advances in anesthesia and analgesia, a significant proportion of patients still suffer from moderate to severe pain after surgery, yet treatments and interventions for these patients are lacking [26, 27]. According to previous reports, the incidence of moderate to severe postoperative pain can be up to 66% in the United States [1]. In particular, orthopedic patients have a higher incidence of postoperative pain. In a study of 10,008 patients in Canada who underwent surgery, the incidence of acute postoperative pain was highest in orthopedic patients [28]. In this study, 41.3% of orthopedic surgery patients under general anesthesia experienced moderate to severe postoperative pain.

Early identification of patients who underwent orthopedic surgery under general anesthesia with a high risk of moderate to severe acute postoperative pain is helpful for early intervention and improving analgesic effect. There are currently no models for predicting postoperative pain in the patient of orthopedic surgery under general anesthesia, while generic postoperative pain prediction models do not accurately predict the degree of acute pain after orthopedic surgery. In addition, risk factors reported in some studies varied widely [8, 10, 13–15, 29, 30] (Table 3). In this study, we constructed a reliable risk prediction model with high discriminatory ability, which is helpful in building personalized treatment plans for patients with an increased risk of acute postoperative pain.

**Table 3:** The risk factors of moderate to severe acute postoperative pain in previous studies

Study (year)	Country	Number of patients	Type of surgery	Risk factors / protective factors
Vasilopoulos et al. (2021)	USA	360	mixed surgery	younger age, female gender, higher anxiety, and more pain behaviors.
Sun et al. (2020)	China	1164	thoracic surgery	younger age, high BMI, preoperative pain, smoking history, and number of chest tubes.
Abrecht et al. (2019)	USA	126	orthopaedic surgery	temporal summation of pain, high BMI, number of previous knee surgeries, and female gender.
Zaslansky et al. (2018)	International	14,334	orthopaedic surgery	female gender, younger age, high BMI, chronic pain, and opioid use before surgery.
Hartwig et al. (2017)	International	192	gastric surgery	younger age and preoperative pain.
Borges et al. (2016)	Brazil	1062	cesarean section	preoperative anxiety, intrathecal morphine with fentanyl^#^.
Liu et al. (2012)	USA	897	orthopedic surgery	female gender, younger age, high BMI, preoperative pain, preoperative use of opioids, general anesthesia, preoperative use of anti-convulsants and anti-depressants, and prior surgery at the surgical site.

# represents the protective factors

Most characteristics of orthopedic surgery patients between group P and group N were significantly different, so it is possible to use them to predict the clinical outcomes. Several risk prediction models were constructed to predict acute postoperative pain by typically performing univariate regression followed by multivariate logistic regression, resulting in reduced prediction accuracy. As an ensemble learning algorithm for classification, random forest is performed by constructing numerous decision trees at training time and outputting the class that is the mode of the classification of the individual trees. Compared with multivariate logistic regression, the random forest algorithm has higher accuracy in classification or prediction tasks and does not require strict assumptions about raw data [31, 32]. We applied the mlr3 package in R to establish and validate a random forest-based prediction model, which has a high ability to handle a multitude of input variables and evaluate the missing data to maintain the prediction accuracy [33].

In this study, the results of ROC analysis showed that the random forest algorithm-based prediction model had higher predictive accuracy than the logistic regression-based model in both the training and the testing sets. To our knowledge, this study is the first attempt to use random forests to predict acute postoperative pain severity in patients undergoing orthopedic surgery under general anesthesia. Our findings demonstrate the potential of random forest algorithms in predicting acute postoperative pain.

In this study, the results demonstrated that the duration of surgery, and blood loss during surgery were significantly associated with acute postoperative pain, which may be related to surgical complexity or surgical trauma size. Abrecht et al. [29] used temporal summation of pain (TSP) to predict postoperative pain accurately. Duration of surgery and blood loss during surgery may be reflections of TSP [34]. Some studies suggest that acute postoperative pain is mainly related to patients rather than surgical factors [13]. In contrast, our study found that postoperative pain was associated with surgical and anesthesia factors. In addition, the use of tourniquets during surgery, indwelling urinary catheters, and arteriovenous catheterization reflects pain from multiple causes other than surgical procedures [35, 36], all of these factors have the potential to predict postoperative pain severity. The above findings remind anesthesiologists that for surgeries that involve large tissue damage and a long operation time, they should pay attention to the dose of analgesics during and after the operation to ensure adequate analgesia. In addition, attention should be paid to the side effects of using tourniquets, indwelling urinary catheters, and drainage tubes.

In previous studies, it has been reported that preoperative pain can increase the incidence of acute postoperative pain [37]. In this study, two factors namely immobilization before surgery and secondary surgery in a short period of time caught the attention. These factors are related to the preoperative pain experience, immobilization is generally used in patients with fractures, and secondary surgery in a short period of time may indicate recently experienced pain. The ability of these two factors to predict postoperative pain has not been reported and can be further investigated in the future. These risk factors alert anesthesiologists to potential pain factors before surgery.

Time from withdrawal of medicine to awake defines the period of time from the cessation of the general anesthetic infusion to the time when the patient becomes conscious. This characteristic was extracted from the patient’s electronic anesthesia records based on our clinical experience. As far as we know, it has not been used in other studies so far. In this study, the characteristic was found to be an important risk factor or predictor of moderate to severe acute postoperative pain. In general, insufficient intraoperative analgesia leads to earlier awakening [38], so we speculate that this characteristic may reflect the adequacy of intraoperative analgesia and may be a potential predictor of acute postoperative pain. This important finding also reminds anesthesiologists to pay attention to adequate intraoperative analgesia.

Currently, many previous studies reported that some demographic characteristics were associated with moderate to severe acute postoperative pain in patients undergoing orthopedic surgery, such as sex, age, and BMI [8, 15, 29]. In this study, after the univariate screening, age was entered into the multivariate logistic regression model and random forest model, younger age was identified as an independent risk factor. However, studies have found that factors such as age are associated with only statistically significant but not clinically significant associations with postoperative pain [39]. In this study, after the univariate screening, sex and BMI were not entered into the models. Therefore, female and high BMI were not included as independent risk factors in this study, which differed from the results of some other studies [8, 15, 29]. We suspect that female and high BMI were widely recognized as risk factors for postoperative pain, timely perioperative interventions, such as multimodal analgesia, were introduced. Therefore, the difference in sex and BMI between group P and group N was not significant.

For the risk factors identified in this study, orthopedic surgeons, anesthesiologists, and nurses need to focus on these factors in their daily work and effectively intervene to reduce acute postoperative pain. The real value of this model is that it can comprehensively evaluate the impact of many variables on outcomes and overcome the limitations of single risk factors. In real-world practice, outcome prediction can be achieved by entering the specific values of each variable included in the model, thereby helping doctors take timely intervention measures for high-risk patients. In the future, the prediction model can be packaged into applications with the help of computer science and other related knowledge, making clinical applications more convenient.

Some limitations of this study are worth mentioning. First, our study was retrospective. In our study, we included as many variables as possible, however, there were still a few characteristics that were not included, such as smoking and drinking habits. Therefore, some valuable factors may not be included. Further studies are needed to investigate whether adding these variables could improve the accuracy of the prediction model. Second, the datasets in our study were collected from a single center and were not large enough. Further studies with large multi-center samples are needed. Last, as a real-world clinical study, the postoperative management of these patients employed different methods for pain management, which varied depending on the patient’s condition, likely contributing somewhat to the variability in pain scores between individuals. Therefore, the risk factors screened out in this study need to be verified by rigorous RCT studies in the future.

## Conclusions

This study addresses the high incidence of acute moderate to severe postoperative pain in orthopedic surgery patients under general anesthesia. We successfully developed a robust predictive model, utilizing the random forest algorithm, which demonstrated strong discriminatory power. The model holds the potential to aid healthcare professionals in early intervention and personalized pain management strategies for orthopedic surgery patients. In addition, this study identified some risk factors that have not been reported in the past and deserve attention in future clinical work.

## Data Availability

The datasets used and analyzed during this current study are available from the corresponding author on reasonable request.

## List of abbreviations

ASA: the American Society of Anesthesiologists
AUC: area under the receiver operating characteristic curve
BMI: body mass index
CI: confdence interval
DCA: decision curve analysis
EMR: electronic medical record
Lasso: the least absolute shrinkage and selection operator
OR: odds ratio
PCIA: patient-controlled intravenous analgesia
RCT: randomized controlled trial
ROC: receiver operating characteristic
SD: standard deviations
TRIPOD: the transparent reporting of a multivariable prediction model for individual prognosis or diagnosis
TSP: temporal summation of pain
VAS: visual analogue scale
WHO: World Health Organization
